# Effect of surface treatments on the bond strength of CAD/CAM fiberglass posts

**DOI:** 10.4317/jced.54904

**Published:** 2018-06-01

**Authors:** Paula-Pontes Garcia, Rogério-Goulart da Costa, André-Vivan Garcia, Carla-Castiglia Gonzaga, Leonardo-Fernandes da Cunha, Carlos-Eduardo-Edwards Rezende, Gisele-Maria Correr

**Affiliations:** 1 Graduate Student, Graduate Program in Dentistry, Universidade Positivo, Rua Pedro Viriato Parigot de Souza, 5300, Curitiba, PR, Brazil; 2 Professor, Dental Prosthesis, Federal Institute for Science and Technology of Paraná, Rua João Negrão 1258, Curitiba, PR, Brazil; 3 PhD Professor, Graduate Program in Dentistry, Universidade Positivo, Rua Pedro Viriato Parigot de Souza, 5300, Curitiba, PR, Brazil

## Abstract

**Background:**

There is no ideal protocol for the surface treatment of fiber posts, especially when using a computer-aided design/computer-aided manufacturing (CAD/CAM) experimental fiberglass block. The purpose of this study was to evaluate the bond strength of a CAD/CAM customized glass fiber post and core after applying different surface treatment techniques.

**Material and Methods:**

Forty premolars were prepared to receive a customized CAD/CAM glass-fiber post and core obtained from an experimental block of glass fiber and epoxy resin. The specimens were randomly distributed in 4 groups (n=10) according to the post and core surface treatment: ETH - 70% ethanol; HP - 24% hydrogen peroxide for 1 minute; ETH/S - 70% ethanol + silane; HP/S - 24% hydrogen peroxide + silane. The universal adhesive containing silane was applied on the posts and prepared post spaces in all groups. The posts were cemented using dual cure resin cement. The specimens were stored in distilled water at 37°C for 24 h, cut (two slices of 1 mm for each root third - coronal, middle, and apical) and subjected to push-out test (0.5 mm/min). Data was subjected to two-way ANOVA (surface treatment and root third) and Tukey’s test (α=0,05).

**Results:**

There was no significant difference of bond strength values among groups, regardless the surface treatment (*p* >0.05). There was significant difference on bond strength values for the different root thirds (*p*<0.05) (coronal>middle=apical).

**Conclusions:**

The different surface treatment and application of additional silane in the CAD/CAM customized glass-fiber post and core does not interfere on bond strength values. The root dentin third interfered on the bond strength, with higher values for the coronal third.

** Key words:**Post and core technique, cad/cam, shear strength, hydrogen peroxide.

## Introduction

The successful rehabilitation of endodontically treated teeth depends on the amount of the remaining tooth structure, the condition of the supporting tissues, the aesthetics of the restoration and reconstruction of the intrarradicular retainer. Posts are often indicated in full crown restorations of non-vital teeth (1). The function of these systems is to promote retention and stability for future prosthetic restoration, which will replace the lost coronary structure (2-5).

The posts are classified according to the materials in metal, ceramic, carbon fiber and glass fiber (6). According to the literature, there is a high success rate in cases treated using glass fiber posts, due to post characteristics, such as modulus of elasticity (about 20 GPa) that is similar to the root dentin elastic modulus (about 18 Gpa) (6). Thus, there is a uniform load distribution and absorption of stresses along the root, decreasing the risk of fractures (1,3).

Other aspect related to the success of indirect restorations is the post adaptation. The need to obtain posts with better adaptation in the root canal led to the introduction of customized posts (7,8). These posts are obtained by direct technique, shaping of the root canal with composite resin associated with pre-fabricated glass fiber post or through the indirect technique, using the dye cast to produce the post in the laboratory. The customization of glass fiber posts allows better adaptation to the prepared post space, which enables the formation of a thin layer of cement, creating favorable conditions for retaining the post (9).

With the improvement of dental computer-aided design/computer-aided manufacturing (CAD/CAM) systems and materials available, CAD/CAM post customization can become an option in dental practices. Studies with clinical follow-up have shown good results in the use of customized posts (10). However, there is no scientific evidence supporting the use of customized CAD/CAM posts to restore endodontically treated teeth, especially regarding the longevity of this treatment.

Post retention depends on the post-cement-dentin bonding and it is estimated that 60% of the fractures occur between post and resin cement (3,11). The bond strength is influenced by several factors, including the shape (12) and the post surface treatment (13).

Glass fiber posts with flat surfaces are a challenge for the mechanical retention. Commonly, adhesive failure is recorded at the interface between the resin cement and post (2). Various surface treatment protocols of glass fiber posts (micro-mechanical or chemical) are reported in the literature to improve the post retention characteristics such as roughening with diamond burs (14), sandblasting with aluminum oxide particles (3), 57.1% ethanol (15), 37% phosphoric acid application (16), 10% hydrofluoric acid (17), and 24% hydrogen peroxide solutions (13,16).

The treatment with hydrogen peroxide has shown good bond strength results of the glass fiber posts. Hydrogen peroxide removes the epoxy resin of the posts and partially exposes the glass fibers without damaging the post structure, and leaving the fibers available to be silanized (3,6,16,18,19-22).

Different surface treatments are often followed by silane coupling agent application. Studies report that the bond strength between posts and resin cements can be improved with the use of silane due to improved wettability (2), and the chemical bond between the monomer matrix of resin cements and the exposed glass fibers (4).

Based on the literature, although there are different protocols for post surface treatment, alternatives to improve the bond between the adhesive interfaces has been constantly studied, especially with the introduction of new technologies and materials. Thus, the aim of this study was to evaluate the effect of different surface treatments on the push-out bond strength of customized CAD/CAM glass fiber posts.

The hypotheses of the study are: i) there is no difference in the bond strength of customized CAD/CAM glass fiber posts to dentin according to the post surface treatment; ii) there might be regional differences in bond strength values between cervical, middle and apical thirds of the root.

## Material and Methods

This study was carried out only after approval by the Institutional Review Board (IRB) of Positivo University (protocol #536.356). Forty intact human maxillary and mandibular single-rooted premolars with anatomically similar root segments and fully developed apices were selected, cleaned in a 0.5% Chloramine T solution for 1 week, and stored in distilled water at 4oC until they were used (no longer than 2 months after extraction).

The roots of the teeth were sectioned 1 mm below the cement-enamel junction (CEJ) to remove all coronal portions and prepared to a 13 mm length. The roots were endodontically treated by the same operator, using a rotary instrument ProTaper Universal (Dentsply Maillefer, Ballaigues, Switzerland). After irrigation and drying, the root canals were filled using gutta-percha and endodontic cement AHPlus (Dentsply Maillefer, Ballaigues, Switzerland) using the Tagger hybrid technique. Root canal openings were filled with a glass ionomer cement (Ionofil Plus; VOCO, Germany), and the samples were stored at 37°C in distilled water for 1 week. Then, the gutta-percha was removed using Gates-Glidden burs, leaving an apical seal of 4 mm.

All groups had the flared post spaces enlarged by removing the internal dentin with a tapered bur, simulating thin-walled canals with a width of 2.5 mm at the occlusal surface, 1.5 mm at the apex, and 9 mm deep measured from the region where the root was cut to apex, to standardize the root canal length.

**Figure 1:**
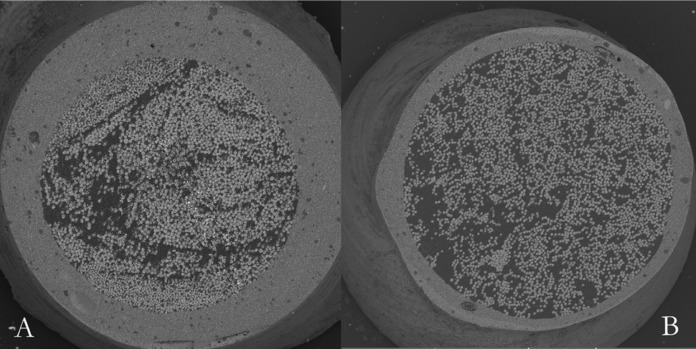
Transversal cut of a pre-fabricated fiberglass post (A) and of the customized CAD/CAM fiberglass post (B), showing similar glass fiber distribution.

For this study, the glass fiber post and core were obtained by a CAD/CAM system from an experimental block of glass fiber and epoxy resin. This block has a similar composition and distribution of the glass fibers of a prefabricated post (WhitePost, FGM, Joinville, Brazil) (Fig. 1).

The glass fiber posts were made from the modeling of the roots using a pattern in self-curing acrylic resin (Duralay, Polidental Cotia, SP, Brazil). The resin pattern was digitized with a 3-dimensional bench top scanner (Ceramill Scanner map400, Ammanngirrbach, Koblach, Austria). The data were processed by the CAD software (Ceramill Mind, Ammanngirrbach, Koblach, Austria). A 3D digital model of the post and core was developed. The experimental block of glass fiber and epoxy resin was milled with a milling machine (Ceramill Motion 2, Ammanngirrbach, Koblach, Austria).

The specimens were then randomly distributed into 4 groups (n=10) according to post surface treatment ( Table 1).

**Table 1:**
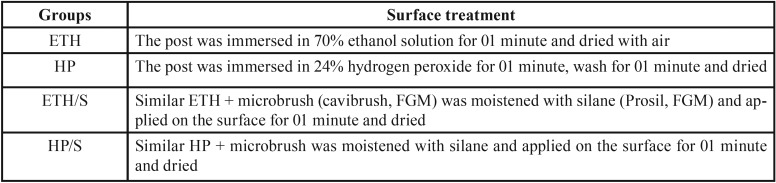
Description of the CAD/CAM glassfiber post surface treatment.

After the surface treatment, the post cementation procedure was equally performed for all groups. First, the universal adhesive containing silane (Single Bond Universal, 3M ESPE, St. Paul, MN, USA) for was applied for 20 s on the glass fiber post surface and dried for 5 s with air. Then, one drop of the adhesive, and a universal optimizer drop (Single Bond Universal OPC) were mixed for 5 s. The mixture was actively applied of the root canal for 20 s, dried for 5 s. The excess of the adhesive was removed with a disposable brush, and was light cure for 10 s at 1.100 mW/cm2 using a LED-curing unit (Poly Wireless, Kavo, Joinville, SC, Brazil). The posts were then luted using dual cure resin cement (Rely X ARC, 3M ESPE, St. Paul, MN, USA), handled according to manufacturer instructions and applied on the post surface with a disposable brush and also in the root canal by a paste carrier tip. All posts were slowly seated by finger pressure, and excess of luting cement was removed with a disposable brush. Once the post was luted, the cement was light-cured from the top of the post with a LED-curing unit for 60 seconds (1100 mW/cm2). Finally, the specimens were stored in distilled water at 37oC for 24 hours.

Before sectioning, each specimen was embedded into acrylic resin blocks (Jet, Artigos Odontológicos Clássico Ltda, São Paulo, SP, Brazil) in a rectangular tube and then transversally sectioned using a water-cooled low-speed diamond saw (Isomet 1000, Buehler, Lake Bluff, IL, USA), resulting in 1.0-mm-thick slices from the coronal, middle, and apical root regions. Diameter and thickness measurements were obtained by using a digital caliper (Mitutoyo, Santo Amaro, SP, Brazil) with 0.01-mm accuracy.

Each slice was subjected to a push-out bond strength test using a universal testing machine (EMIC DL 2000 São José dos Pinhais, PR, Brazil) at a crosshead speed of 0.5 mm/min. The load was applied in the apical-coronal direction until the dislodgment of the post. For all tests, the push-out pin on the center of the post surface was carefully placed and different sizes of punch pins were used to match the diameter of the post at the different root thirds being tested.

**Figure 2:**
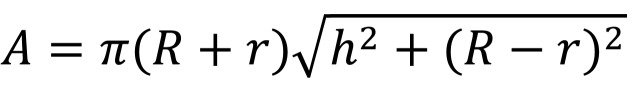
Formula.

The maximum failure load was recorded in Newtons (N) and converted into MPa by dividing the applied load by the bonded area. The bonded area was the lateral area of a truncated cone, which was calculated by the formula: (Fig. 2).

where A is the lateral area of a truncated cone, π = 3.14, R = coronal post radius, r = apical post radius, and h = root slice thickness.

The failure modes were evaluated at 57x magnification under a stereomicroscope (SZX9, Olympus, Tokyo, Japan). Failure was assessed and classified as follows: 1) adhesive between post and resin cement (no resin cement visible around the post); 2) adhesive between resin cement and root dentin (resin cement covering the post); 3) mainly cohesive within the resin cement; 4) mixed. Representative specimens of each group were selected and observed under SEM.

Also, SEM micrographs were taken to visualize the CAD/CAM post and core surface characteristics after the different surface treatments.

Mean values and standard deviations were calculated for each group. Data were subjected to two-way ANOVA considering the factors surface treatment and root third, and to Tukey’s test (α=0.05). All the statistical analyses were performed using Statistica version 10.0 (StatSoft South America Comércio de Software LTDA, São Caetano do Sul, SP, Brazil).

## Results

Means (in MPa) and standard deviations of the push-out bond strength values for the different groups are presented in  Table 2.

**Table 2:**
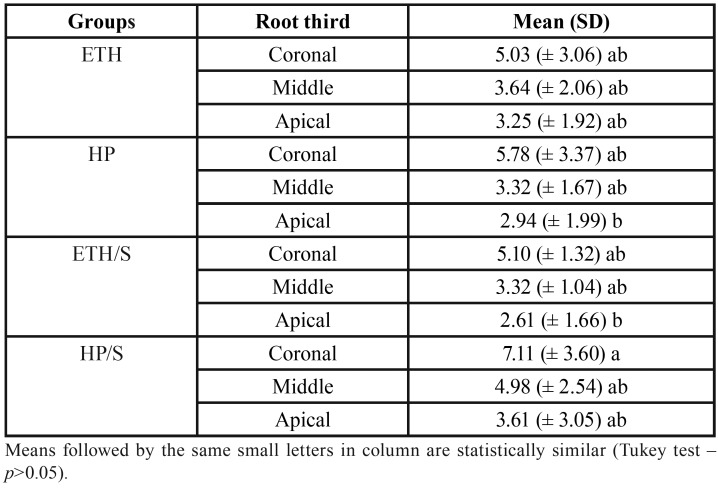
Means (in MPa) and standard deviation after push out test for the different groups (n=10).

According to analysis of variance, there was no significant difference among surface treatments (*p*=0.104006) and for the interaction treatment x root third (*p*=0.906978). There was a significant difference among the root canal thirds (*p*=0.000052). Thus, a multiple comparison analysis by Tukey test was performed.

The higher push-out bond strength values were observed for the group treated with hydrogen peroxide followed by silane application (HP/S) in the coronal third, differing from groups HP and ETH/S, both in the apical third, that showed the lowest bond strength values.

Considering the individual factors, the surface treatment did not showed significant difference on bond strength values, regardless the root canal third. Regarding the root canal third, there was a significant difference on the bond strength values, as follows: Coronal (5.76±3.13 MPa)>Middle (3.72±2.07 MPa)=Apical (3.2±2.46 MPa). The higher values were observed in the coronal third that differ from the other thirds.

**Figure 3:**
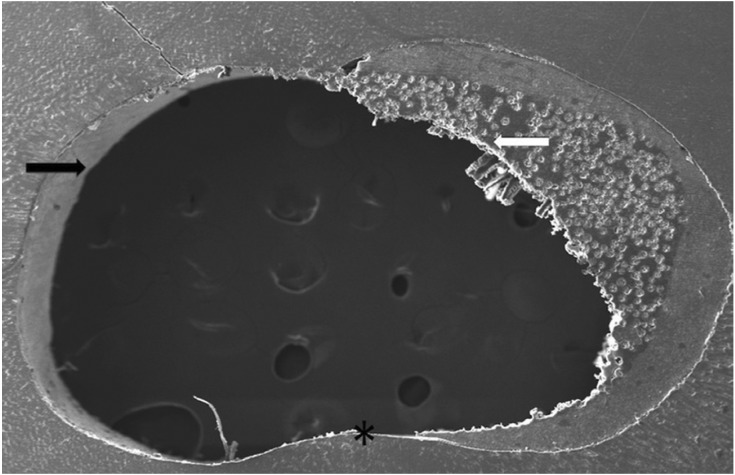
Representative SEM image of the most frequent type of failure found in all groups, mixed failure (combination of adhesive between resin cement and root dentin (asterisk), adhesive between resin cement and post (black arrow), and cohesive in the fiberglass post (white arrow).

The most frequent failure mode found for all groups was mixed (combination of adhesive between resin cement and root dentin, adhesive between resin cement and post, and cohesive in the glass fiber post), as shown in Figure 3.

**Figure 4:**
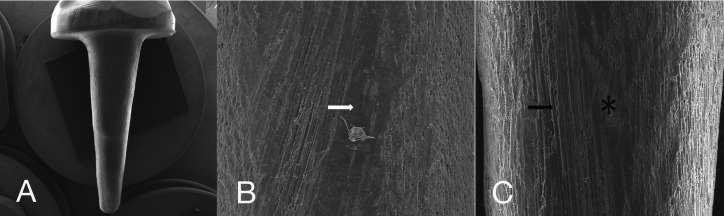
SEM micrograph of the customized CAD/CAM glass fiber post and core design (A), and of the surface characteristics after ethanol application (B) or hydrogen peroxide (C). The white arrows show the epoxy layer covering the fibers and the black arrows show the exposed glass fibers. Asterisks shows the areas where damage to fiber integrity has occurred, caused by the CAD/CAM milling procedure.

The SEM micrographs of the CAD/CAM glass fiber post and core and its surface characteristics after the different treatments are shown in Figure 4. The customized glass fiber post and core shows a uniform design with round edges. For both treatments (ethanol and hydrogen peroxide) the post surface characteristics are similar, showing a rough surface with some exposed unidirectional glass fibers. In hydrogen peroxide groups, the exposed glass fibers are more apparent.

## Discussion

The success of restorative treatment also depends, among other factors, on the bond strength between the post and the tooth structure. Therefore, the post shape, its adaptation to the root canal, and the adhesive and cement used play an important role in the treatment success (23).

In order to improve the post adaptation, various post customization techniques have been proposed. The most common technique is the use of composite resin covering a prefabricated glass fiber post (1). However, this technique has the disadvantage of the creation of another adhesive interface between post and composite resin, increasing the chances of failures. Additionally, contamination among increments of the resin or between post/resin interface can occur. In this study, a new method to obtain the customized post and core was proposed, with the use of an experimental block of glass fiber and epoxy resin in a CAD/CAM system. One of the advantages of CAD/CAM system is the fabrication of the post and core in a single piece, without the creation of interfaces between fiber post and composite resin (10,24), what could be seen in Figure 4A. Also, the customized CAD/CAM glass fiber post and core used in this study showed similar characteristics, such as good quantity and distribution of glass fibers, when compared to a commercially available pre-fabricated glass fiber post (White post, FGM, Joinville, SC, Brazil), as showed in Figure 1. However, when using CAD/CAM systems with post, the optimal protocol for ensuring clinical success requires further investigations because many systems, softwares and scanners are available. Thus, the adaptation can be dependent of these system variables.

Another approach to provide better retention of glass fiber post is the surface treatment (3,11-13). The main objective of the different post surface treatments is to increase the bond strength to resin cement. Fiber posts are composed of unidirectional glass fibers embedded in an epoxy resin matrix. The epoxy matrix is substantially incapable of reacting with the monomers of the resin cement. Thus, the post surface treatment becomes essential, because it will partially remove the epoxy resin matrix, exposing the glass fibers so that they can receive the silanization (13,16,18,20).

The literature reports various surface treatments, namely chemical, mechanical or chemical-mechanical. What should be taken into consideration is that many substances used in the surface treatment can damage the post fibers, such as phosphoric acid and hydrofluoric acid at high concentrations and micro-mechanical treatments with abrasive particles, thus hampering the bond strength (13,16,18).

In this study four post and core surface treatments (alcohol or hydrogen peroxide, with or without silane) were proposed. According to the results, there was no significant difference between groups, corroborating findings in the literature (16,20). Thus, the first hypothesis was accepted.

The effect of hydrogen peroxide treatment on adhesion is based on the oxidation of the post surface, partially removing the epoxy resin (21), and exposing the glass fibers to silanization and bonding to the resin cement. The effective use of hydrogen peroxide as surface treatment of glass fiber post is well documented in the literature (3,16,18-22).

In this study, it was used 24% hydrogen peroxide for 1 minute. It was reported that this protocol, concentration and time, is capable to partially remove the epoxy resin layer of the post, exposing the glass fibers without any damage to post structure (18). However, in this study there was no statistical difference among the surface treatments. As could be seem in the SEM images, the post surface characteristics after ethanol or hydrogen peroxide treatments are similar. Both showed exposed glass fibers. Thus, it can be suggested that the machining itself, in the CAM system, could have exposed the glass fibers by removing the excess of epoxy resin.

In addition to the different treatments with the application of ethanol or hydrogen peroxide, the application of the silane coupling agent in the post surface has been evaluated in this study. The function of the silane coupling agent was to promote a chemical bond between the glass fibers and the resin based cement and it is essential in enhancing the hydrolytic stability of the glass fiber post/resin cement interface (24). Thus, after removing the epoxy matrix, the glass fibers are exposed and ready to be silanized.

In addition to the silane agent applied after surface treatment with ethanol or hydrogen peroxide, it was used, in this study, a universal adhesive system (Single Bond Universal), which has silane in its composition. There is little information in the literature about the use of this adhesive system on bond strength of glass fiber posts.

The effect of the incorporated silane in the bonding agent (Single Bond Universal) on the repair of CAD/CAM resin and ceramic blocks was also evaluated in a study (24). The authors noted that depending on the surface treatment performed in the CAD/CAM block, etching with hydrofluoric acid or tribochemical treatment, only the silane present in the adhesive was not enough to promote an adequate bond strength. However, when it was performed a macro-mechanical treatment, wear with diamond bur, the application of an additional layer of silane did not affect the cement bond strength to ceramic block. This is in accordance with the results found in this study, where the application of an additional layer of silane did not affect bond strength values. In this case, the surface treatment of the glass fiber post (application of hydrogen peroxide) may not have promoted significant changes in the post microstructure and hence the silane present in the adhesive system was sufficient to cause adequate bond strength (24).

In this study, there was a significant difference among the root thirds, with higher bond strength values in the coronal third compared with the other thirds. Thus, the second hypothesis was accepted. This is due to greater penetration of light during the light curing in this region, since the depth of cure decreases gradually towards the apex (16). However, it can be observed in this study there was no significant difference between the bond strength values in the middle and apical thirds. The use of a self-etching adhesive system (Single Bond Universal) together with the polymerization optimizer (OPC) and dual cure resin cement allow a good bond strength in apical third, where light penetration is lower.

The most common type of failure found in this study was mixed, followed by adhesive failure. Considering the post-resin cement-dentin interface, the mixed failure is considered more favorable when compared to the adhesive failure, because it proves an adequate seal between post and dentin. It was observed that, regardless of the surface treatment used, the bonding procedure was effective, thus confirming the results found in the literature (20,24,25).

According to the literature, there is not an ideal protocol for surface treatment of fiber posts, especially for new materials such as CAD/CAM experimental glass fiber block. Based on the results of this study, for this type of post, simple procedures, which require less clinical time and have greater technical practicality, such as cleaning with 70% alcohol and the application of a universal adhesive are indicated.

Different methods can measure bond-strength between endodontic posts and the tooth structure. In the present study, the push-out test was used because in this test the shear stresses occurs at dentin/cement and post/cement interfaces (11) and it has been used in many studies 

It can be concluded that the different surface treatments of the customized CAD/CAM glass fiber post did not interfere in bond strength values. The root third region influences the bond strength values, which was higher in the coronal third.
